# Dermal Papilla Cell-Derived Extracellular Vesicles Increase Hair Inductive Gene Expression in Adipose Stem Cells via β-Catenin Activation

**DOI:** 10.3390/cells11020202

**Published:** 2022-01-07

**Authors:** Taheruzzaman Kazi, Abir Nagata, Takatoshi Nakagawa, Takashi Matsuzaki, Shigeki Inui

**Affiliations:** 1 Department of Regenerative Dermatology, Graduate School of Medicine, Osaka University, Osaka 565-0871, Japan; abir.med@osaka-u.ac.jp (A.N.); tnakagawa@r-derma.med.osaka-u.ac.jp (T.N.); inui@r-derma.med.osaka-u.ac.jp (S.I.); 2 Department of Biological Science, Faculty of Life and Environment Science, Shimane University, Shimane 690-0823, Japan

**Keywords:** dermal papilla cells (DPCs), extracellular vesicles (EVs), dermal papilla cell-derived EVs (DPC-EVs), adipose-derived stem cell (ASCs), differentiation, β-Catenin, FGF2 and PDGF-AA

## Abstract

Recently, extracellular vesicle (EV)-mediated cell differentiation has gained attention in developmental biology due to genetic exchange between donor cells and recipient cells via transfer of mRNA and miRNA. EVs, also known as exosomes, play a role in maintaining paracrine cell communication and can induce cell proliferation and differentiation. However, it remains unclear whether adipose-derived stem cells (ASCs) can adopt dermal papilla (DP)-like properties with dermal papilla cell-derived extracellular vesicles (DPC-EVs). To understand the effect of DPC-EVs on cell differentiation, DPC-EVs were characterized and incubated with ASCs, of monolayer and spheroid cell cultures, in combination with the CAO1/2FP medium specialized for dermal papilla cells (DPCs). DPC-like properties in ASCs were initially evaluated by comparing several genes and proteins with those of DPCs via real-time PCR analysis and immunostaining, respectively. We also evaluated the presence of hair growth-related microRNAs (miRNAs), specifically mir-214-5P, mir-218-5p, and mir-195-5P. Here, we found that miRNA expression patterns varied in DPC-EVs from passage 4 (P4) or P5. In addition, DPC-EVs in combination with CAP1/2FP accelerated ASC proliferation at low concentrations and propagated hair inductive gene expression for versican (*vcan*), alpha-smooth muscle actin (*α-sma*), osteopontin (*opn*), and N-Cam (*ncam*). Comparison between the expression of hair inductive genes (*vcan*, *α-sma*, *ctnb*, and others), the protein VCAN, α-SMA and β-Catenin (CTNB), and hair inductive miRNAs (mir-214-5P, mir-218-5p, and mir-195-5p) of DPC-EVs revealed similarities between P4 DPC-EVs-treated ASCs and DPCs. We concluded that early passage DPC-EVs, in combination with CAP1/2FP, enabled ASCs to transdifferentiate into DPC-like cells.

## 1. Introduction

Hair loss or alopecia is the aftereffect of inefficacy in regrowing hair shafts from hair follicles (HFs) in both males and females. Hormonal perturbance and extrafollicular environmental factors affect follicular stem cell properties, resulting in the loss of HFs [1]. To prevent hair loss, prescribed medications and hair transplantation are typical measures, but so far they are not able to revive HFs. The HF, an integumentary organ, has a unique structure that contains numerous concentric epithelial keratinocytes and a specialized mesenchymal cell called a dermal papilla cell (DPC) [2]. These specialized mesenchyme-derived cells are aggregated at the lower end of the HF. DPCs maintain HF development, hair cycle activity, and hair shaft growth [3,4]. In the case of HF morphogenesis, DPCs intricately interact with the surrounding epithelial cells via a paracrine mechanism [5]. A recent study revealed that EVs released from cells play a role in cell–cell interactions as paracrine factors [6]. EVs carry microRNA (miRNA) and transcription factors to the nearby cell niche to regulate the functions of the cells [7]. In recent years, the use of miRNA to induce stem cell differentiation and regulate the post-transcriptional expression of target genes has gained attention [8]. 

Stem cells maintain tissue niches through self-renewal, differentiation of multi-lineage cells, and through the secretion of various types of growth factors [9]. For therapeutic options, induced-pluripotent stem cells (iPSCs), embryonic stem cells (ESCs), and adult stem cells are widely used owing to their pluripotency and auto-reproducibility, in spite of the limitations of the two former types (iPSCs and ESCs) owing to ethical considerations and genetic manipulation [10,11,12]. In contrast, adult stem cells have no ethical issues to be considered. Mesenchymal stem cells (MSCs) are adult stem cells that are mainly obtained from the bone marrow stroma; however, the harvesting procedure is highly invasive and the differentiation potential declines with age [13]. Adipose stem cells (ASCs), with similar cell morphology and differentiation potential as bone marrow-derived MSCs, are isolated from white adipose tissue [14], which can be easily collected. White adipose tissue accumulates at various specific locations called adipocyte depots, which play definite cellular and molecular functions [15,16]. A considerable quantity of adipocyte depots is subcutaneous, while others are visceral and located in the abdominal cavity [17]. The upper layer of the subcutaneous tissue is referred to as an intradermal adipocyte depot, consisting of adipose stem/precursor cells. These cells communicate with each other via growth factors, such as platelet-derived growth factor (PDGF), during HF development and hair cycle, especially in the anagen phase of postnatal development [18]. In addition, ASCs can differentiate into many other cell types—that is, adipogenic and osteogenic—under appropriate differentiation media. There are several types of molecular markers that distinguish these two cell types; the typical expression markers are leptin for adipogenic cells and osteopontin for osteogenic cell. Notably, these two markers are also expressed in DPCs, in a hair cycle-dependent manner.

DPCs are derived from mesenchymal cells, and both DPCs and mesenchymal stem cells have a similar hair induction-related gene expression, such as the leptin (*Lep*) and osteopontin genes [18,19]. The lineage-specific development and differentiation of stem cells largely depends on the in vivo cellular microenvironment [20]. To induce lineage-specific differentiation of stem cells, including iPSCs, ESCs, MSCs, and ASCs, specific media mimicking the in vivo microenvironment are required [21]. The aim of the study was to characterize DPC-EVs and examine the possible role of DPC-EVs in the differentiation of ASCs into DPC-like cells. This was achieved by analyzing miRNA expression in combination with a specialized medium to imitate the DPC microenvironment.

## 2. Materials and Methods

### 2.1. FBS EV Depletion and EV Isolation

To avoid contamination, FBS was filtered with a 0.22 µm filter (Merck, Burlington, MA, USA), followed by ultracentrifugation at 120,000× *g* for 18 h in a fixed-angle rotor 70ti (Beckman Optima L-90K, Beckman Coulter, Brea, CA, USA). FBS-derived EVs were pelleted at the bottom of the tube, and the supernatant was collected as EV-depleted FBS. For EVs isolation from the culture medium, B6-DPCs were cultured in a 100 mm dish up to 70–80% confluence. The cells were washed three times with phosphate-buffered saline (PBS) and then cultured in DMEM/10% FBS (EV-depleted) for 48 h. Serial centrifugations were conducted at 4 °C from cell pellet removal to EV purification. First, a 10 min 300× *g* centrifugation was used to eliminate the cell pellet, then centrifugation at 2000× *g* for 20 min was employed to eliminate the dead cells. To eliminate cell debris and large particles, centrifugation was performed at 10,000× *g* for 30 min. Thereafter, the supernatant was collected and non-gradient ultracentrifugation was performed at 100,000× *g* for 70 min. For EV purification and elimination of protein contamination, supernatant was gently removed. The pellet was resuspended in cold PBS, and ultracentrifugation at 100,000× *g* was performed for 70 min. After resuspension, the EVs were preserved at −80 °C.

### 2.2. Transmission Electron Microscope (TEM)

TEM analysis was used to confirm the presence of DPC-EVs in the conditioned medium. For fixation, equal volumes of EVs and 4% paraformaldehyde were absorbed by formvar carbon-coated EM grids for 30 min and washed twice with PBS for 3 min. The grid was rinsed 4 times with 50 mM glycine in PBS for 3 min, then blocked with Blok-Ace for 30 min and incubated with anti-CD63 for 1 h at 1:100 ratio. The antibody was washed with PBS and 0.05% Tween-20 (T-PBS) for 3 min and treated with 10 nm gold particles conjugated with a secondary antibody for 30 min, followed by incubation with 1% glutaraldehyde for 5 min. After washing with T-PBS several times, the EVs were observed by TEM at 80 kV. For the negative control, all the above procedures were followed except for primary antibody incubation. EVs were only incubated with 1% glutaraldehyde for 5 min. EVs size distribution or diameter was measured by ImageJ software (public domain free software version: java 1.8.0_172) after capture of EV images by TEM. Total Area(A) of EVs was measured upon nanometer (nm) scale. Later, the following equation was used for EVs diameter (D = nm) analysis.

$$A\left(nm\right)=\pi r^{2},\;r=\sqrt{\left(\frac{A}{\pi }\right)},\;D=2r\;(\mathrm{nm}).$$

### 2.3. Cell Culture 

#### 2.3.1. Culture Media

CAO1/2FP was prepared as previously reported [22]. Briefly, Dulbecco’s modified Eagle’s medium (DMEM) with 10% FBS (DMEM-FBS) adipogenic and osteogenic media were mixed in a 1:1 ratio and addressed as a combination of adipogenic and osteogenic media (CAO). The final concentrations of adipogenic and osteogenic inducers in CAO were: 550 nM dexamethasone (MP Biomedicals, Santa Ana, CA, USA; 1000 nM for adipogenic and 100 nM for osteogenic media), 0.5 µM IBMX (Wako, Osaka, Japan; adipogenic media), 1 μg/mL insulin (Sigma-Aldrich, St. Louis, MO, USA; adipogenic media), 10 mM β-glycerol phosphate (TCI, Tokyo, Japan; osteogenic media), and ascorbic acid 50 µg/mL (Wako, Osaka, Japan; osteogenic media). When the CAO was diluted twice with the DMEM-FBS, it was named CAO1/2. Subsequently, CAO1/2 was supplemented with FGF2 and PDGF-AA growth factors (PeproTech, Rocky Hill, NJ, USA) at 100 ng/mL; this culture medium was named CAO1/2FP. To evaluate the effect of DPC-EVs on ASCs, EV-depleted FBS was administrated in all experiments.

#### 2.3.2. Isolation of DPCs

DPs were isolated from the C57BL/6-mouse vibrissa follicles in the anagen phase and then cultured in papilla cell growth medium (PCGM; Toyobo, Japan) at 37 °C with 5% CO_2_. Outgrowing cells and DPCs were collected using Accutase^TM^ (Innovative Cell Technologies, San Diego, CA, USA) and sub-cultured to propagate up to passage 4 (*p* = 4), then aliquoted and preserved in liquid N_2_.

#### 2.3.3. Isolation of ASCs

Mouse adipose tissue was harvested from subcutaneous fat and inguinal fat pads, extensively washed with PBS, and finally minced. To remove clotted blood, muscle, or skin debris from the minced tissue, the fragments were centrifuged for 10 min at 300× *g* in a 50 cc conical tube. Floating tissue fragments were digested for 60 min at 37 °C in collagenase (Worthington Chemicals, Lakewood, NJ, USA) at a final concentration of 0.1%, while gently shaken. The digestion was stopped with DMEM-10% FBS, and the remaining samples were gently collected and filtered through a 100 mm cell strainer (BD Biosciences, San Jose, CA, USA). The cell supernatant was washed twice for 10 min and centrifuged twice for 5 min at 300× *g*. The pellets were resuspended in DMEM-10% FBS containing 1% penicillin/streptomycin (Merck, St. Louis, MO, USA), and cells were counted. The cells were plated at a density of 1 × 10^6^ cells in 100 mm culture dishes at 37 °C with 5% CO_2_. After 24 h, non-adherent cells were removed. The adherent cells and ASCs were cultured with up to 80–90% confluence and passaged once with trypsin/EDTA (Thermo Fisher Scientific, Amarillo, TX, USA). For ASCs, passage 2 cells were used in the experiment.

Thawed ASC aliquots were cultured in CAO1/2 FP medium in a 6-well culture plate at a density of 7 × 10^4^ cells/well. DPC-EVs were added to the culture medium on day three. On day six, the cells were dissociated with 0.25% trypsin/EDTA and inoculated in a low-adhesive 96-well plate (Prime surface; Sumitomo Bakelite, Tokyo, Japan) at 7 × 10^3^ cells/well to form a three-dimensional (3D) structure (spheroid) over 3 days.

**Scheme 1:**
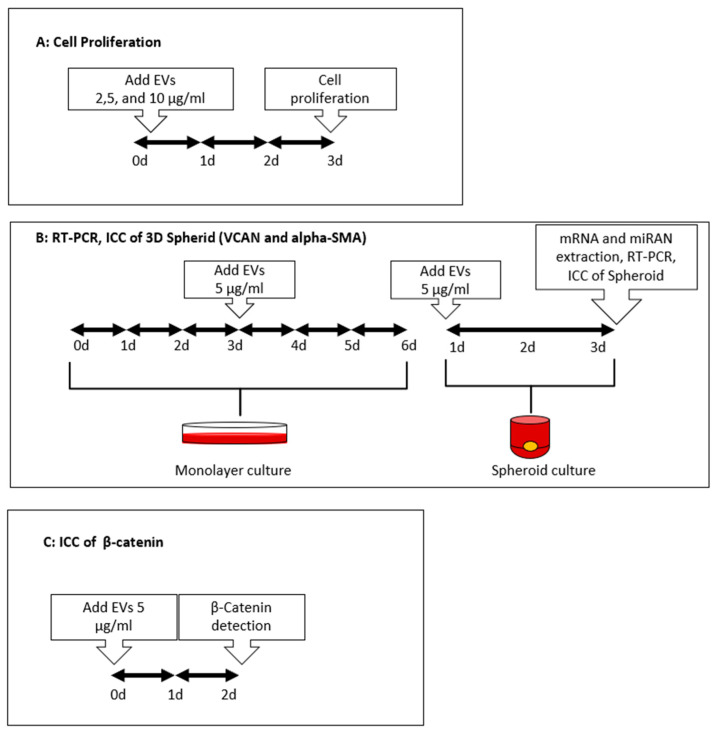
The schematic figure represents experimental design.

Unless stated otherwise, the ASCs in this study were cultured as spheroids. Spheroid culture was used since it is deemed more similar to in vivo models and important for maintaining stemness properties [23]. ASCs in a spheroid culture system were inoculated with 5 µg/mL of DPC-EVs (Scheme 1).

The EV uptake assay was performed to ensure that the EVs were biologically active. EV uptake by ASCs was evaluated by incubating the cells with DPC-EVs for 48 h and then staining them with anti-CD63 antibodies to detect the incorporated EVs. 

### 2.4. Oil O Red and Alkaline Phosphatase (ALP) Staining

To understand the capability of inducing differentiation of the CAO1/2 medium, ASCs were co-stained with Oil O red and ALP. Briefly, cells were fixed with 4% paraformaldehyde (PFA) for 10 min, washed in PBS, and then incubated in the substrate solution NBT/BCIP and TN Buffer for 30 min, in accordance with the manufacturer’s instructions (Roach, Basel, Switzerland). The cells were then washed in the culture plate with Milli-Q water, rinsed with 60% isopropanol for 1 min, and stained with 0.3% Oil O red (Merck, St. Louis, MO, USA) for 30 min, rinsed with 60% isopropanol, and finally washed with Milli-Q water three times. For nuclei staining, the stained cells were incubated with 0.1% methyl green for 30 s.

### 2.5. Immunocytochemistry

The expression of *VCN* and *ASMA* was also confirmed by immunocytochemical analysis of spheroids (3D). DPC and ASCs with DPC-EVs spheroids were stained with anti-versican or α-SMA antibodies, followed by secondary antibodies conjugated with appropriate fluorescence labeling. To detect CTNB and PDGFa expression, ten thousand ASCs were inoculated in LAB-TEK II chamber slides (8 wells/slide; Thermo Fisher Scientific, Franklin Lakes, NJ, USA) for 2 and 7 days, respectively. The cells were then fixed with 4% PFA for 10 min and permeabilized with 0.1% Triton X-100 for 10 min at room temperature (25 °C). They were then blocked for 30 min with Block Ace (DS Pharma Biomedical, Osaka, Japan) in accordance with the manufacturer’s instructions and treated with anti-α-SMA antibody (MAB1420, R&D Systems, MN, USA), anti-PDGF-a polyclonal antibody (NBP2-99113 AF647, NOVUSBIO, Littleton, CO, USA), mouse anti-versican antibody (NBP2-22408 MAB, NOVUSBIO, Littleton, CO, USA), and anti-CD63 antibody (EPR21151, Abcam, Cambridge, UK) for the EV uptake assay. They were thoroughly washed with PBS containing 0.05% Tween-20 and reacted with secondary antibodies conjugated with Alexa Fluor 594 (ab150116), Alexa Fluor 647 (ab150115), and Alexa Fluor 488 (ab150113 and ab150077 for EVs), all conjugated with the secondary antibodies at a 1:600 dilution. DAPI Fluoromount-G (Southern Biotech, Birmingham, AL, USA) was used for nuclei staining. Fluorescent images were collected under a BZX-800 fluorescence microscope (Keyence, Osaka, Japan) and viewed with another fluorescence microscope (BX51N, Olympus, Shinjuku City, Tokyo, Japan), for Figures S1 and S2, with a digital monochrome camera DFC345FX (Leica Microsystems, Wetzlar, Germany, DE; all experiments related to Figures S1 and S2 were performed at Shimane University).

### 2.6. mRNA Extraction and RT-PCR

Total RNA from the spheroids of each category was collected on day three using the RNeasy Mini Kit (Qiagen, Hilden, Germany). cDNA was prepared from the RNA using the QuantiTect reverse transcription kit (Qiagen, Venlo, The Netherlands). Real-time PCR analysis was performed to examine the gene expression levels (Table 1) using the Thermal Cycler Dice^®^ Real-Time System (TP 950; Takara Bio, Shiga, Japan) and cDNA template combined with the TB-Green Premix Ex Taq II kit (RR820A, Takara Bio, Shiga, Japan). β-actin was used as an internal control in all experiments.

### 2.7. miRNA Extraction and RT-PCR

Total miRNA was collected from EVs and/or ASC EVs using the miRNeasy Mini Kit (Qiagen, Hilden, Germany). cDNA was prepared using the TaqMan™ MicroRNA Reverse Transcription Kit (Thermo Fisher Scientific, Waltham, MA, USA). The expression levels of hsa-mir-218-5p (assay ID: 000521), hsa-mir-214 (assay ID: 002293), hsa-miR-195 (assay ID: 000494), and internal control U6snRNA (assay ID: 001973) (Table 2) were analyzed using the Thermal Cycler Dice^®^ Real-Time System (TP 800; Takara Bio, Shiga, Japan) and the TaqMan Fast Universal PCR Master Mix (2X) (4366072, Thermo Fisher Scientific, Waltham, MA, USA).

### 2.8. Protein Concentration and Western Blot

Dermal papilla cells and EVs were lysed in RIPA buffer (182-02451, Fujifilm, Japan). Protein concentrations were measured using a BCA protein assay kit (Thermo Fisher Scientific, Waltham, MA, USA). Extracted proteins from cells and EVs resolved in 10% SDS-PAGE were electrotransferred onto nitrocellulose membrane by semi-dry system. Then the blots were incubated in 0.3% BSA in TBST (Trish buffer saline with 0.1% Tween-20) for 1 h in room temperature. Specific primary antibodies (Rabbit mAb anti-CD63 #ab217345, rabbit mAb anti-TSG101#ab125011, rabbit mAb anti- beta Actin #ab115777, rabbit pAb anti-Hsp90#ab13495, and rabbit pAb anti-CYC1#ab137757) were added to the blots at 1:1000 dilution and incubated in 4 °C for overnight. The blots were washed in TBST and incubated in appropriate secondary antibody-conjugated horseradish peroxide (ab97051, Cambridge, UK). The blots were developed using chemiluminescence reagent (Luminata #WBLUR0500, Darmstadt, Germany).

### 2.9. Cell Proliferation Assay

MTT Cell Proliferation Assay Kit (Ab-211091, Cambridge, UK) was used for the cell proliferation assay. Manufacturer’s instructions were followed. Briefly, the cell proliferation assay was performed at day three after EV inoculation in cell culture medium. The culture supernatant of adherent cells was carefully aspirated from the 12-well culture plate (TPP, Harlev, Denmark). A 1:1 ratio of serum-free media and MTT reagent mixture was added into each well. Subsequently, the culture plate was incubated at 37 °C for 3 h. After incubation, 1.5 volume of MTT solvent was added into the mixture in each well. After 15 min of orbital shaking, the culture plate was read with a plate reader (SH-9000lab, Corona Electrical, Hitachinaka-shi, Japan). Absorbance was measured at OD = 590 nm. The cell number of the sample plate was evaluated by comparison with the known cell number of the standard curve.

### 2.10. Statistical Analysis

We performed a one-way analysis of variance (ANOVA) with post hoc Tukey HSD test (online web statistical calculator, https://astatsa.com/OneWay_Anova_with_TukeyHSD/) for multiple groups, while Student’s *t*-test was used for comparing two groups (Microsoft Excel).

## 3. Results

### 3.1. Identification of DPC-EVs

**Figure 1:**
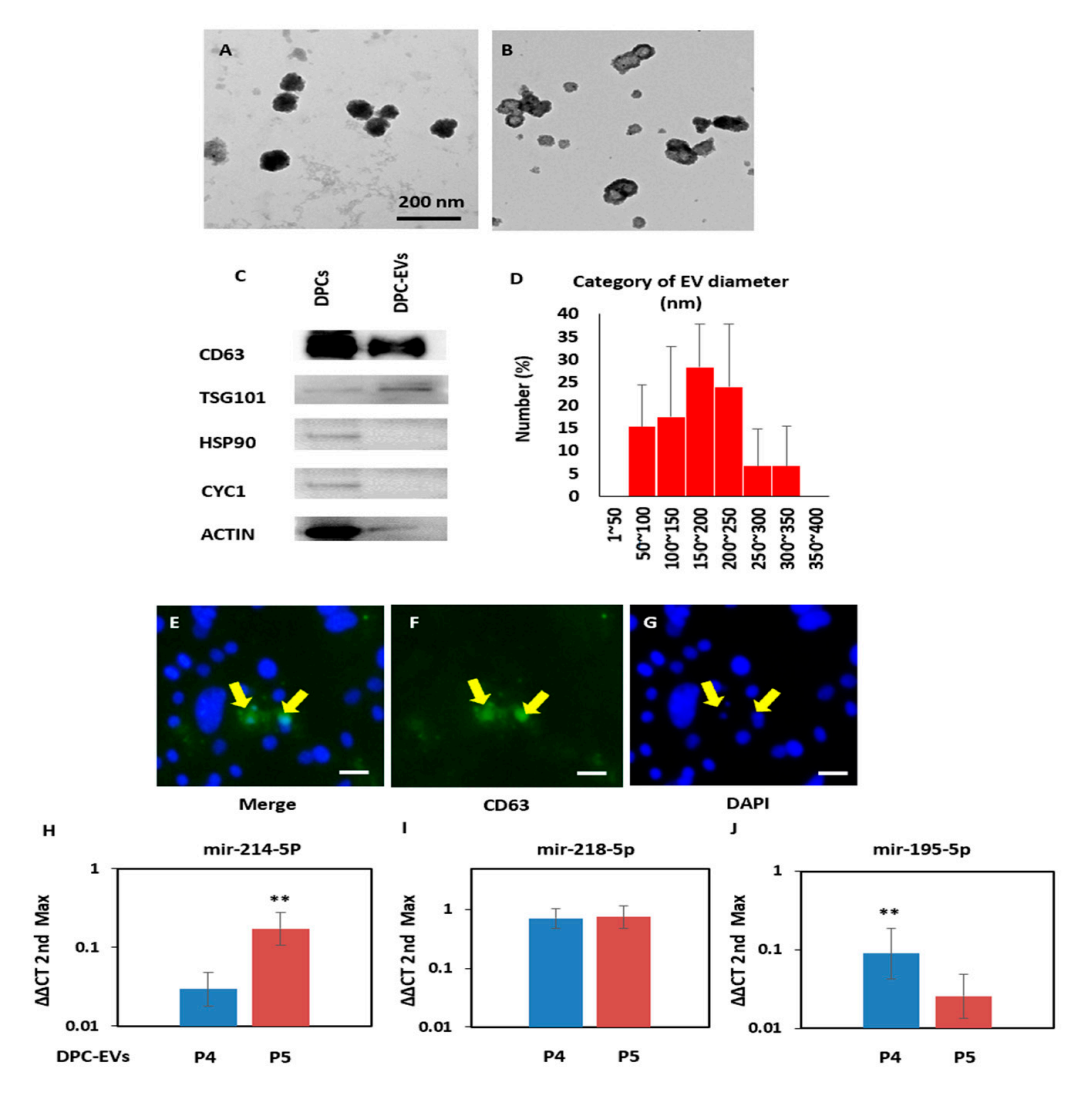
Isolation and detection of dermal papilla cell-derived extracellular vesicles (DPC-EVs) by transmission electron microscopy (TEM) and quantitative PCR (qPCR) analysis of miRNA expression in DPC-EVs. (**A**) DPC-EVs were detected by EV-specific anti-CD63 antibody followed by secondary antibodies conjugated with 10 nm gold beads (dense, black appearance). (**B**) As a negative control, DPC-EVs were stained with 1% glutaraldehyde, and no gold beads were attached. (**C**) DPCs and EVs were scrutinized by Western blot with specific antibodies. (**D**) The size distribution DPC-EVs were analyzed by ImageJ software 1.8.0_172. (**E**–**G**) Uptake of DPC-EVs by adipose-derived stem cells (ASCs). The yellow arrow indicates the EVs uptaken by ASCs; scale bar indicates 10 µm. (**H**–**J**) TaqMan-based real-time PCR analysis of exosomal mir-214-5P, mir-218-5p, and mir-195-5P in P4 and P5 DPC-EVs (*n* = 3). Data are presented as the mean value ± SD (Error bars). ** *p* < 0.01.

To investigate the role of DPC-EVs, they were collected from DPCs, as described in the Materials and Methods section. Transmission electron microscopy (TEM) analysis confirmed the presence of DPC-EVs in the conditioned medium, which was stained with an anti-CD63 surface marker conjugated with secondary antibodies and 10 nm gold particles (Figure 1A). No signal was detected in samples treated with 1% glutaraldehyde only (Figure 1B). Western blot analysis revealed the presence of ubiquitous EV markers, such as CD 63 and TSG101 (tumor susceptibility gene 101) protein, which are enriched in DPC-EVs compared with cell lysates (DPC). On the other hand, HSP90 (heat shock protein 90) and CYC-1 (cytochrome C), which belong to cell fractions, were depleted from EV (Figure 1C). The TEM images analysis indicates the average diameter of EVs was between 80 nm and 170 nm (Figure 1D). We performed an EV uptake assay to ensure that the EVs were biologically active. EV uptake by ASCs was evaluated by incubating the cells with DPC-EVs for 48 h and then staining them with anti-CD63 antibodies to detect the incorporated EVs (Figure 1E–G). CD63-positive signals were detected in the peri- and intra-nuclear areas of the incubated ASCs. We also examined the hair-related regulatory miRNAs—mir-214-5P, mir-218-5P, and mir-195-5P—in EVs derived from passage 4 (P4) or passage 5 (P5) DPCs. The keratinocyte proliferation inhibitor mir-214 was 5.3 times lower in P4 DPC-EVs than P5 DPC-EVs (Figure 1H), which was significant. The CTNB activator mir-218-5P was similarly detected in P4 and P5 DPC-EVs (Figure 1I). The CTNB regulatory mir-195-5P was 3.5 times higher in P4, suggesting significance (Figure 1J).

Taken together, isolated DPC-EVs were purified and biologically active, which was supported by Western blot, the sufficient uptake of ASCs, and the presence of miRNAs.

### 3.2. Induction of Cell Proliferation and Inculcation of Hair Induction-Related Gene Expression in ASCs with DPC-EVs

**Figure 2:**
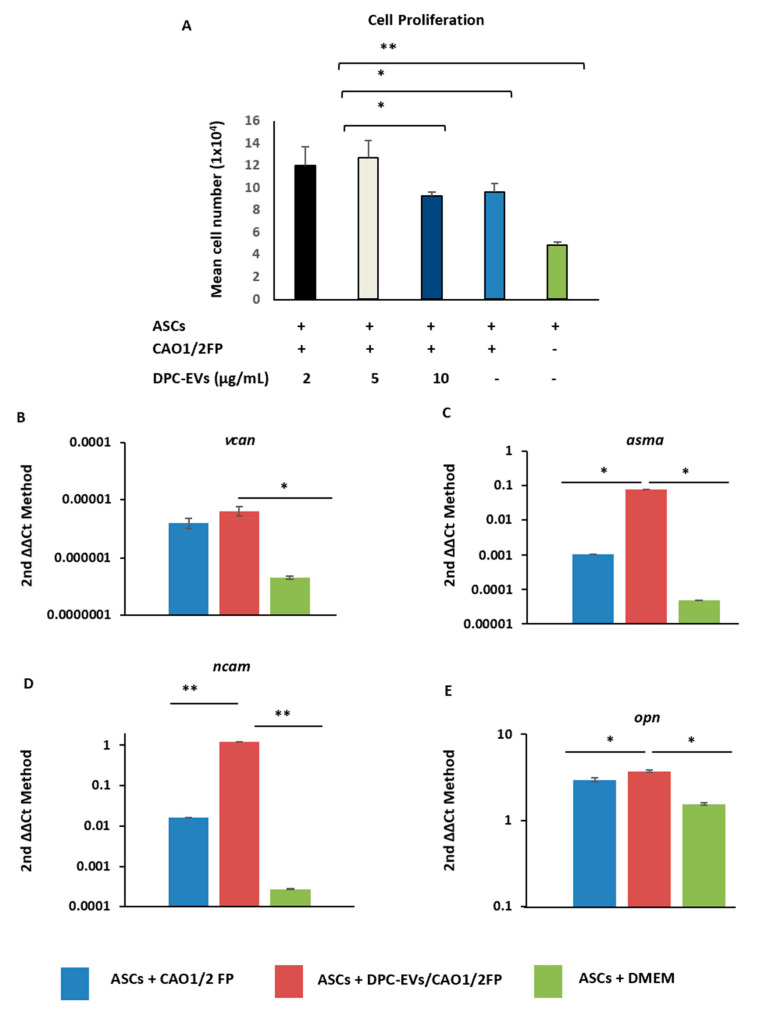
Proliferation and hair-related gene expression of adipose-derived stem cells (ASCs) incubated with dermal papilla cell-derived extracellular vesicles (DPC-EVs) in combination with CAO1/2FP. (**A**) Effect of DPC-EVs on ASC proliferation. (**B**–**E**) Expression of the DPC-related genes in the ASC spheroids. Spheroids were prepared in CAO1/2FP medium ± DPC-EVs or DMEM-FBS. The DPC-related genes, *vcan* (**B**), *asma* (**C**), *ncam* (**D**), and *opn* (**E**), were analyzed with Taqman-based quantitative PCR. The average gene expression for each category was obtained from two independent experiments measured in triplicate, and data are presented as the mean value ± SD (Error bars). * *p* < 0.05, and ** *p* < 0.01.

It has been previously reported that mir-214 overexpression inhibited cell proliferation [24]. To circumvent the excessive influence of mir-214, P4 DPC-EVs, expressing lower mir-214-5P compared with P5 DPC-EVs, were selected for further experiments. First, we examined the effect of DPC-EVs on ASC proliferation. CAO1/2FP increased ASC proliferation compared with DMEM. Surprisingly, 2 and 5 µg/mL and not 10 µg/mL DPC-EVs with CAO1/2FP significantly enhanced cell proliferation compared with CAO1/2FP only (Figure 2A). This suggests that 5 µg/mL of DPC-EVs with CAO1/2 is optimal for cell proliferation and hair inductive gene expression in ASCs. The DPC-EVs expressed mir-195 (Figure 1H). Hair induction-related genes in ASCs were significantly higher in CAO1/2FP than in DMEM, even without DPC-EVs (Figure 2B–E). Real-time PCR analysis revealed that DPC-EVs significantly induced the expression of *vcan*, *ncam*, *α-sma*, and *opn* in ASCs of the spheroid culture system (Figure 2B–E). These data suggest that DPC-EVs with CAO1/2FP (DPC-EVs/CAO1/2FP) were biologically active and could transform ASCs into DPC-like cells with hair formation activity.

### 3.3. Transdifferentiation of ASCs into DPC-like Cells through Incubation with DPC-EVs

**Figure 3:**
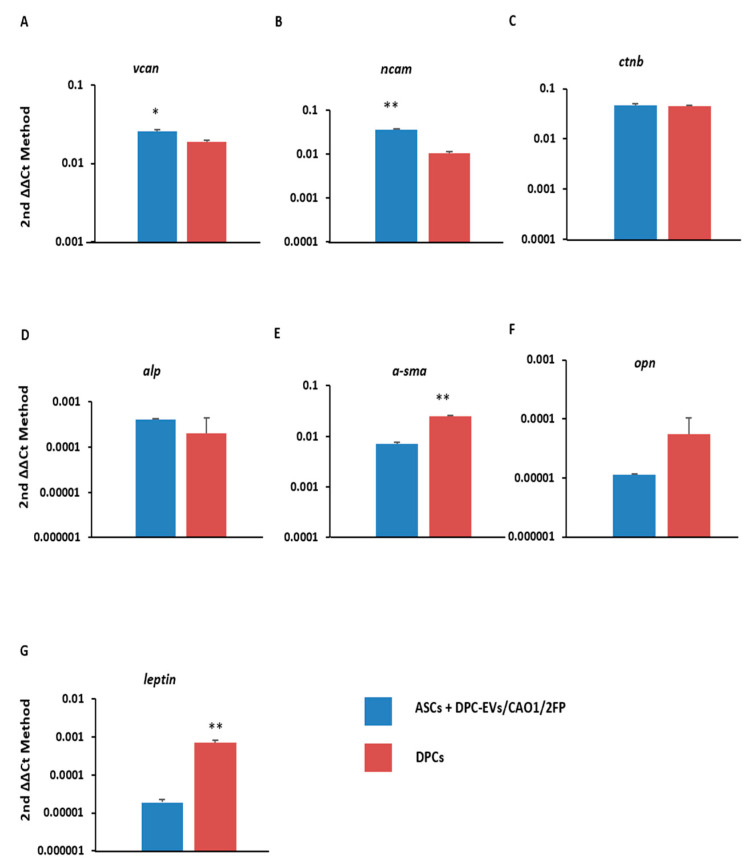
Comparison of hair induction related genes. (**A**–**G**) The gene expression was analyzed with real-time PCR, comparisons are illustrated between adipose-derived stem cells (ASCs) with DPC-EVs and dermal papilla cells (DPCs). Total RNA was isolated from each spheroid. The average gene expression for each category was obtained from three independent experiments measured in triplicate, and data are presented as the mean value ± SD (Error bars). * *p* < 0.05 and ** *p* < 0.01.

**Figure 4:**
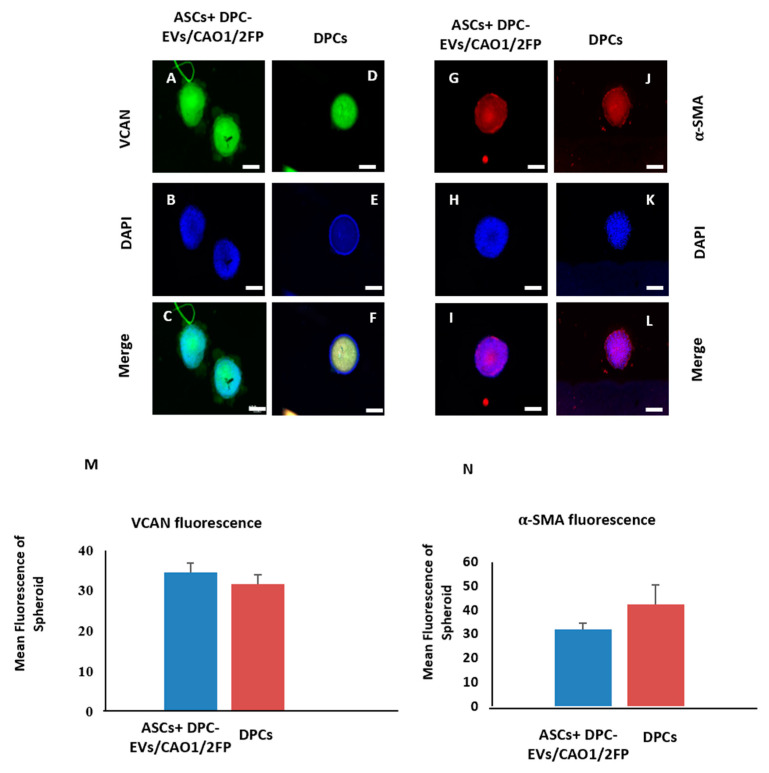
Immunofluorescence microscopic analysis of VCAN and α-SMA in dermal papilla cells (DPCs) and adipose-derived stem cells (ASCs) with dermal papilla cell-derived extracellular vesicles (DPC-EVs) spheroids. (**A**–**L**) Representative photograph of cells in each category, stained with anti- VCAN and α-SMA antibodies. (**M**,**N**) Quantitative histograms of VCAN and α-SMA fluorescence intensity in spheroids were made using the Image J software 1.8.0_172. The white scale bar represents 200 µm. Data are presented as the mean value ± SD (Error Bars) and showed no significant difference between the two groups.

DPCs can regenerate HFs in the mammalian hair cycle and express genes related to hair induction, such as versican (*vcan*), *ctnb*, *α-sma*, alkaline phosphatase (*alp*), *opn*, *ncam*, and anagen inducer leptin (*Lep*). We observed that the expression of vcan and ncam was, respectively, 1.3 and 3.6 times higher in ASCs with DPC-EVs compared with DPCs (Figure 3A,B). The genes ctnb and alp were expressed similarly in both cell types (Figure 3C,D). In contrast, expression of α-sma, opn, and leptin was significantly higher in DPCs than in ASCs with DPC-EVs (Figure 3E–G). The expression of VCAN (Figure 4A–F) and α-SMA (Figure 4G–L) was also confirmed by immunocytochemical analysis of spheroids (3D). DPCs and ASCs with DPC-EVs spheroids were stained with anti-VCN or α-SMA antibodies, followed by secondary antibodies conjugated with appropriate fluorescence labeling. As shown in Figure 4M,N, the expression of both proteins was almost consistent with the mRNA expression (Figure 3A,E). These data further support the ability of DPC-EVs to transdifferentiate ASCs into DPC-like cells.

### 3.4. Induction of the CTNB Pathway in ASCs through Incubation with DPC-EVs and miRNAs

**Figure 5:**
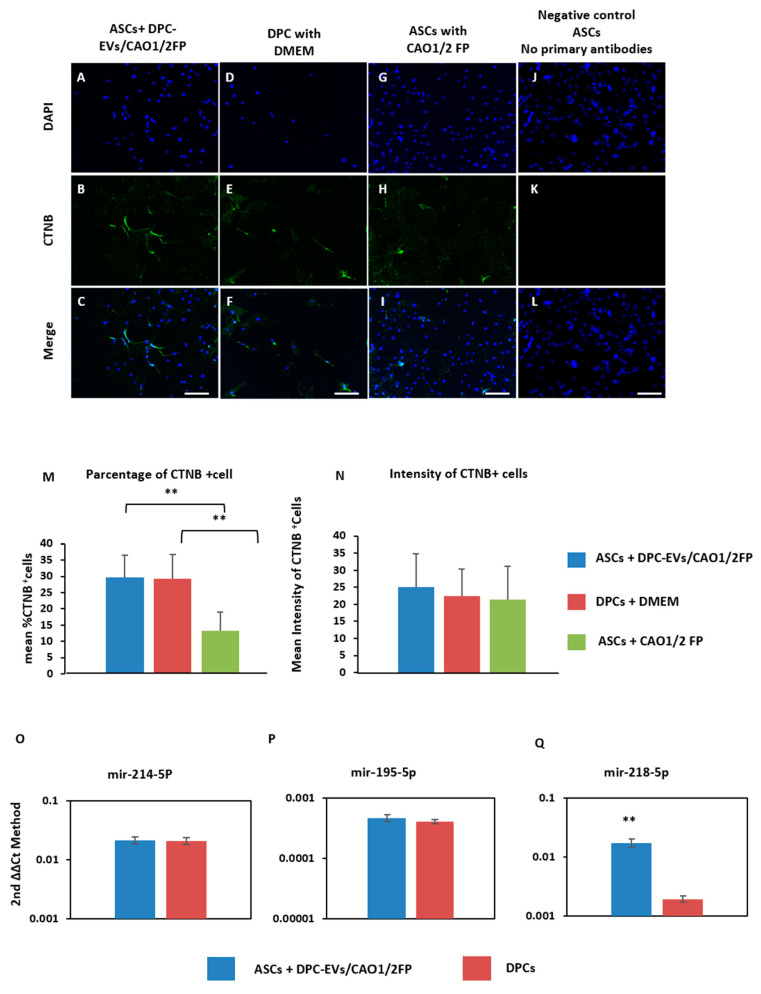
Immunofluorescence microscopic analysis of CTNB in adipose-derived stem cells (ASCs) under various conditions. (**A**–**C**) Immunofluorescence image of CTNB-positive cells in ASCs treated with DPC-EVs/CAO1/2FP, (**D**–**F**) dermal papilla cells (DPCs) with DMEM, and (**G**–**I**) ASCs with CAO1/2FP. (**J**–**L**) Negative control with no primary antibodies. (**M**) Percentage of CTNB-positive cells of total nuclei from randomly selected images. (**N**) Fluorescence intensity of CTNB-positive cells was quantified with Image J software 1.8.0_172. (**O**–**Q**) CTNB regulatory miRNA expression between DPCs and DPC-derived extracellular vesicles (DPC-EVs) treated ASCs with CAO1/2FP, white scale bar represents 100 µm. Vertical bars represent the mean value ± SD (Error bars), ** *p* < 0.01.

CTNB has been implicated as playing a pivotal role in HF development [25]. If DPC-EVs can transdifferentiate ASCs into DPC-like cells, then CTNB will be induced in ASCs via incubation with DPC-EVs/CAO1/2FP. To examine this possibility, we analyzed the expression of CNTB in ASCs and ASCs with DPC-EVs. CNTB was expressed in DPCs, as expected (Figure 5D–F). Monolayer ASCs, not spheroidal, under various conditions were utilized for staining. CTNB-positive cells were observed in ASCs cultured in CAP1/2FP and were also increased by the addition of DPC-EVs (Figure 5A–I,M). No signal was observed in the negative control (Figure 5J–L), and mean fluorescence intensities were consistent among the three groups (Figure 5N). It has been reported that mir-214 and mir-195-5P overexpression inhibits the canonical CTNB signaling pathway [24,26]. To gain further insight into the DPC-EVs/CAO1/2FP-induced transformation of ASCs, we compared the expression of these miRNAs between DPCs and ASCs with DPC-EVs/CAO1/2FP. MicroRNAs were collected as described in Materials and Methods. Mir-214-5P and mir-195-5P were similarly expressed in both cell lines (Figure 5O,P). Notably, mir-218-5p, which has been reported to promote hair growth via upregulation of CTNB signaling [27], was significantly higher in ASCs with DPC-EVs than in DPCs alone (Figure 5Q). These results further suggest that DPC-EVs, in cooperation with CAP1/2FP, can transdifferentiate ASCs via the induction of the CTNB signaling pathway and a certain set of miRNAs—namely, mir-195-5p.

## 4. Discussion

In this study, the properties of DPC-EVs were characterized, and the potential of DPC-EVs and/or DPC-EVs/CAO1/2FP to transfer the properties of DPCs into ASCs was also explored. Our study was composed of three sections: (1) identification and characterization of DPC-EVs, (2) comparison of hair-related/inductive gene expression between DPCs and ASCs with DPC-EVs, and (3) further characterization of ASCs with DPC-EVs by examining the CTNB signaling pathway.

First, we identified DPC-EVs and the hair-related miRNAs, mir-195-5p, mir-214-5p, and mir-218-5p in the DPC-EVs. The miRNA expression in P4 and P5 DPC-EVs was quantified by reverse transcription real-time PCR. As DPC-EVs contained mir-195, we hypothesized that it may enhance the expression of hair-inductive genes in ASCs, as well as increasing cell proliferation. However, it has been reported that miRNA expression changes in in vitro-cultured DPCs in a passage-dependent manner, and a high expression of one such miRNA, mir-195-5p, inhibited DPC proliferation when it was transfected [24]. Consistent with this, we revealed that exosomal miRNA expression varied between cell passage P4 and P5 (Figure 1H–J). In particular, mir-214 was significantly higher in P4 DPC-EVs than in P5 DPC-EVs. As mir-214 overexpression has been reported to be responsible for the reduction in HFs in developing skin, and for a delay in hair cycle progression during postnatal development [26], DPC-EVs derived from the lower passage cells might have increased hair growth activities. Thus, we hypothesized that the lower expression of mir-214 in P4 DPS-EVs implies its potential role in providing DP-like properties to ASCs. In contrast, P4 and P5 DPC-EVs displayed similar mir-218-5p expression (Figure 1I). Hu et al. reported that mir-218-5p in EVs upregulates *Ctnb* and contributes to HF neogenesis [27]. However, P5 and P4 DPC-EVs might affect CTNB signaling and HF neogenesis differently. We have revealed that DPC-EVs, at concentrations as low as 2 μg/mL in combination with CAO1/2FP, enhanced the proliferation of ASCs (Figure 2A). This strongly suggests that DPC-EVs are biologically active and affect the transformation of ASC properties into DP-like properties. Although DPC-EVs at the highest concentration (10 μg/mL) inhibited cell proliferation, high amounts of miRNAs such as mir-214, a known cell inhibitory miRNA, might affect ASC proliferation. We are currently working on the influence of mir-214 on ASC proliferation and differentiation into DPC-like cells.

We previously reported that CAO1/2FP medium (a combination of adipogenic and osteogenic inducers at low concentrations, with FGF-2 and PDGF-AA) was adequate for DPC in vitro culture and prevented DPC shrinkage by maintaining extracellular matrix components, versican, and other hair induction-related gene expression [22]. We used CAO1/2FP in the current study as a differentiation medium for ASCs. The key features of CAO1/2 medium were to prevent ASCs from differentiating into mature adipocytes or osteocytes (Figure S1Q–S) and enable ASCs to express hair-inductive genes such as *vcan*, *α-sma*, and *ctnb* (Figure S1K–P). EVs were endocytosed by recipient cells, and the RNA cargo in the EVs was transferred, which in turn altered the recipient cell gene expression profile and function [28,29]. We analyzed the expression of hair induction-related genes expressed by DPCs in ASCs treated with CAO1/2FP, DPC-EVs/CAP1/2FP, and DMEM. CAO1/2FP seemed to be appropriate for ASCs and DPCs to transform ASCs into DPC-like cells. DPCs express characteristic genes, such as *vcan*, *sma*, *sox2*, *ctnb*, *Alpl*, and so on, for HF morphogenesis into the postnatal hair cycle in mammals [22,30,31]. These genes are also expressed in non-DPCs, such as dermal sheath cells and dermal fibroblasts; however, combinations of these molecules are cell-type specific. For instance, DPCs are α-SMA-positive but DESMIN-negative, whereas dermal sheath cells are DESMIN-positive [29] and MSCs are SOX2-positive [23,32], indicating that DPCs form heterologous populations. Nonetheless, we found that DPC-EVs enhanced *vcan*, *ncam*, and *α-sma* gene expression in ASCs, which are highly specific in the DPCs of anagen stage HFs (Figure 2B–E). When profiles of hair induction-related genes were compared between ASCs with DPC-EVs and DPCs, these were similar (Figure 3A–G). This indicates that DPC-EVs in combination with CAO1/2FP can transform ASCs into DPC-like cells, which is also supported by immunocytochemical analyses of VCAN and ASMA (Figure 4A–N). CAO1/2FP contains FGF-2 and PDGF-AA. FGF upregulates the expression of CTNB via Erk1/2 activation upon receptor binding [33]. In contrast, PDGF-AA is a key molecule secreted as a paracrine factor from DPCs in the dermis, which promotes HF development and hair growth [18]. In this study, we found that DPC-EVs in CAP1/2FP stimulated CTNB expression in ASCs (Figure 5M,N). DPC-EVs might work, but cooperatively with the growth factors in CAP1/2FP. CTNB plays an important role in cell cycle progression, cell proliferation, and gene regulation of *Cyclind1* and *c-Myc*, as well as *Ctnb*. Upon Ctnb activation, it accumulates cytoplasm and translocates into the nucleus [34], and then transcriptionally regulates various events, such as the maintenance of the HF anagen phase [35].

Collectively, we demonstrated that DPC-EVs, especially prepared from P4 DPCs, could transdifferentiate ASCs into DPC-like cells in combination with CAP1/2FP, which was evidenced by the induction of hair growth-related genes via the CTNB pathway. Adipose tissue is rich in ASCs that can be easily collected from excess body fat in a relatively safer manner, compared with the collection of DPCs.

ASC-derived DPC-like cells might be useful in the treatment of hair-related diseases, such as alopecia, either independently or in combination with other available therapies. The limitations of our study, however, are a limited knowledge of miRNA expression, other than the miRNAs under examination, and an insufficient characterization of DPC-EVs in a passage-dependent manner. Further studies will be required to understand the therapeutic potential of DPC-EVs and ASC-derived DPC-like cells.

## Figures and Tables

**Table 1:** Primer of RT-PCR.

Primer Name	Forward Primer (5′-3′)	Reverse Primer (5′-3′)
versican	ACTAACCCATGCACTACATCAAG	ACTTTTCCAGACAGAGAGCCTT
α-smooth muscle actin	GGCACCACTGAACCCTAAGG	ACAATACCAGTTGTACGTCCAGA
alpl	GTGACTACCACTCGGGTGAAC	CTCTGGTGGCATCTCGTTATC
Osteopontine	TGCAAGCCCTTTGGCAATG	GCCGTCCATATAGCAGCCC
leptin	CCAGTTGGTAACAATGCCATGT	TTCAAAGCCGAGGCATTGTTT
beta-catenin (ctnb)	ATGGAGCCGGACAGAAAAGC	CTTGCCACTCAGGGAAGGA
ncam	AGAAATCAGCGTTGGAGAGTCC-	TCGTCATCATTCCACACCACT
CD44	CACCATTGCCTCAACTGTGC	TTGTGGGCTCCTGAGTCTGA
CD45	ACCACCAGGTGAATGTCAATTT	CTTGCTTTCCCTCGGTTCTTT
CD90	TGCTCTCAGTCTTGCAGGTG	TGGATGGAGTTATCCTTGGTGTT
Pdgf-a	ACATGAGGGGACTCTGGGAA	ACAGTGAAGCCCAACAGCTT
Pdgf-b	ATTGTGCGAAAGAAGCCCATC	GGGTCACTACTGTCTCACACTT
beta-Actin	GGCTGTATTCCCCTCCATCG	CCAGTTGGTAACAATGCCATGT

**Table 2:** Taqman microRNA sequences.

miRNA Name	Mature miRNA Sequence
mir-218-5p	UUGUGCUUGAUCUAACCAUGU
mir-214-5p	UGCCUGUCUACACUUGCUGUGC
mir-195-5p	UAGCAGCACAGAAAUAUUGGC
Internal control U6sn	GTGCTCGCTTCGGCAGCACATATACTAAAATTGGAACGATACAGAGAAATTACATGGCCCCTGCGCAAGGATGACACGCAAATTCGTGAAGCGTTCCATATTTT

## Data Availability

All the data analyzed during this study are included in this article. Data are available upon request.

## Supplementary Materials

The following are available online at https://www.mdpi.com/article/10.3390/cells11020202/s1, Figure S1: Surface marker expression of ASCs, and their DP-related gene expression at different combinations of adipogenic and osteogenic factors, Figure S2: Growth factor expression of ASCs stimulated with CAO1/2.
