# Supplementary material for: Dermal Papilla Cell-Derived Extracellular Vesicles Increase Hair Inductive Gene Expression in Adipose Stem Cells via β-Catenin Activation
Source: Cells. 2022 Jan 7;11(2):202. doi: 10.3390/cells11020202 (PMC8773911; doi:10.3390/cells11020202)
Supplement: Supplementary file 1 [file cells-11-00202-s001.zip › cells-1495100-supplementary.pdf]

Surface marker expression of ASCs, and their DP-related gene expression at different combinations of adipogenic and osteogenic factors.

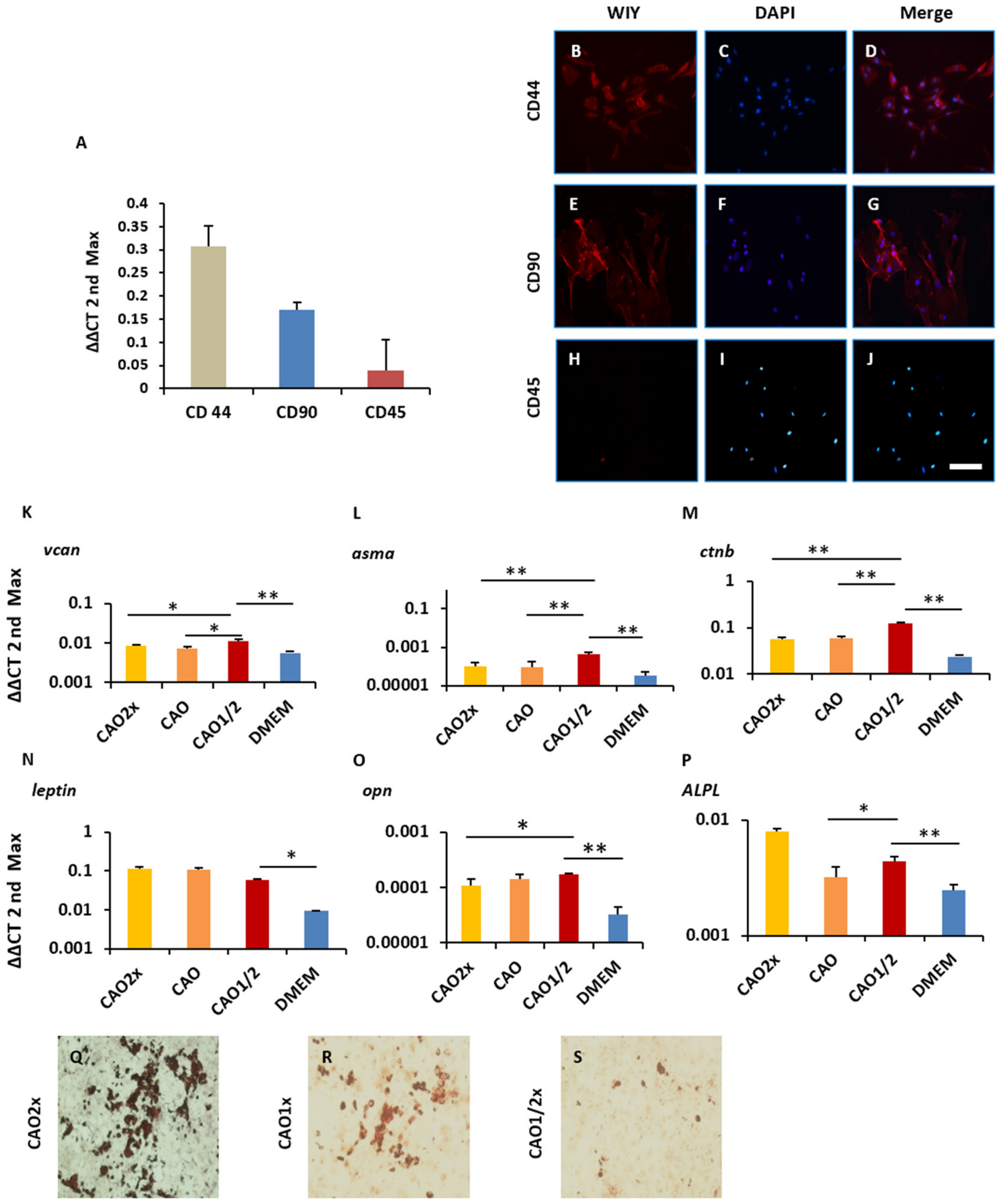

Supplementary Figure S1. Surface marker expression of ASCs, and their DP-related gene expression at different combinations of adipogenic and osteogenic factors. (**A**) gene expression of CD44 and CD90 are positive markers, and CD45 is a negative marker of ASCs. (**B**–**G**) Immunocytochemical expression Positive surface markers of ASCs and (**H**–**J**) negative surface markers, white scale bar represents 100 μm. (**K**–**P**) Hair inductive gene expression observed in ASCs cultured in co-induction media of adipogenic and osteogenic inducers at various concentrations. (**Q**–**R**) Representative photographs of ASCs differentiation into adipocytes and osteocytes induced by Adipogenic and osteogenic inducers at various concentration, (**S**) but no oil droplet was observed in lowest concentration referred as CAO1/2 which were detected by Oil red O and BCIP/NBT co-stein. The mean value ± SD was calculated from the average of triplicate measurements (* $p < 0.05$, ** $p < 0.01$, CAO2x, CAO, CAO1/2 and DMEM).

Growth factor expressions of ASCs stimulated with CAO1/2

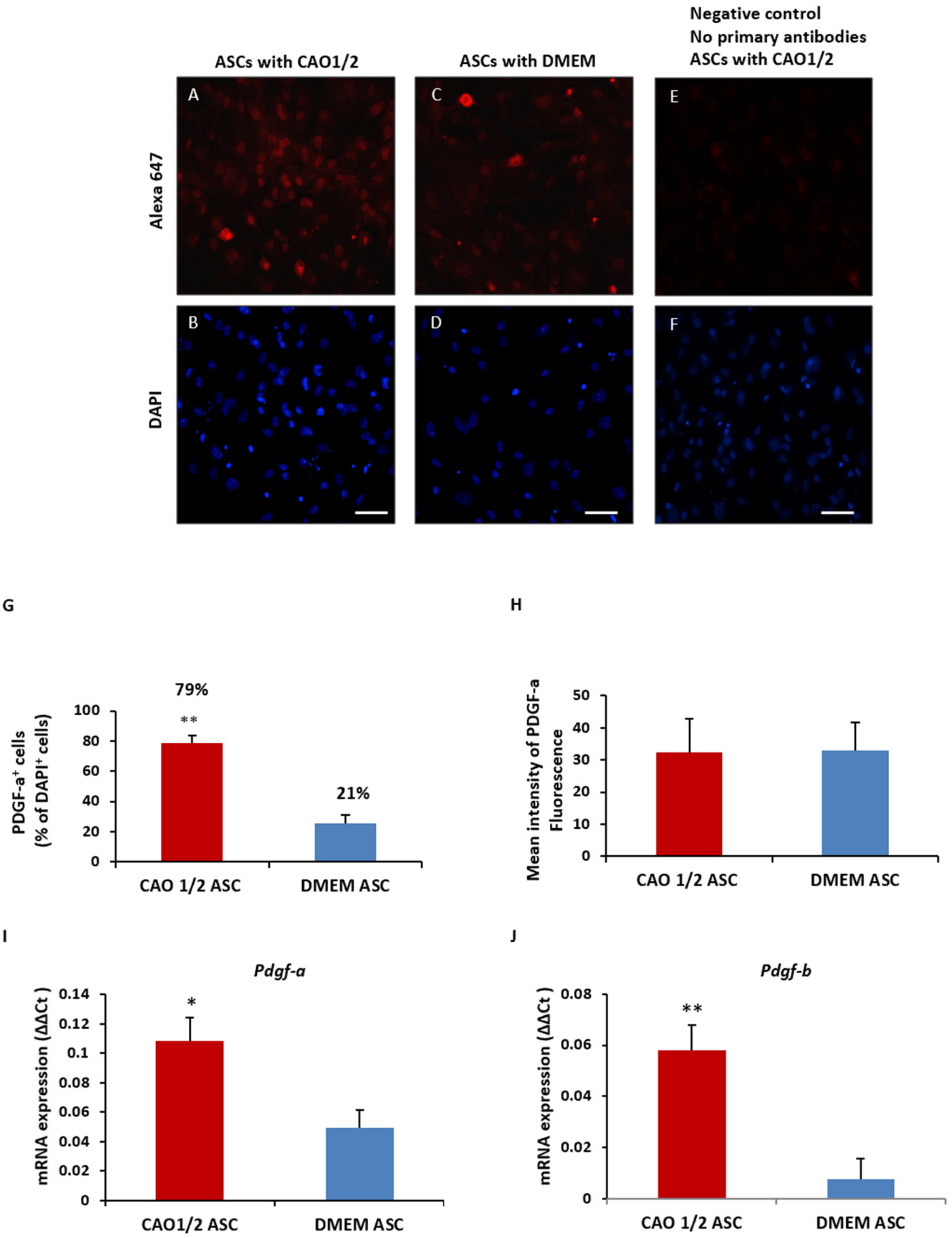

Supplementary Figure S2. Growth factor expression of ASCs stimulated with CAO1/2. (**A** and **B**) Immunocytochemical expression of endogenous PDGF-a in CAO1/2 stimulated ASCs, (**C** and **D**) DMEM-stimulated ASCs, and (**E** and **F**) negative control (no primary antibodies). (**G**) The percentage of PDGF-positive cells were measured from the total nuclei of randomly selected fields. (**H**) The fluorescence intensity of PDGF-a was measured using ImageJ. (**I** and **J**) The mRNA expression of growth factors was quantified by qPCR. The mean value ± SD was calculated from the average of triplicate measurements. White scale bar represents 100μm (* $p < 0.05$, ** $p < 0.01$).
